# Well-Being and Institutional Care in Older Adults: Cross-Sectional and Time Effects of Provided and Received Support

**DOI:** 10.1371/journal.pone.0161328

**Published:** 2016-08-22

**Authors:** Aleksandra Kroemeke, Ewa Gruszczynska

**Affiliations:** Department of Psychology, SWPS University of Social Sciences and Humanities, Warsaw, Poland; Banner Alzheimer's Institute, UNITED STATES

## Abstract

**Background:**

The aim of the study was to examine the cross-sectional and longitudinal effects of provided and received support on older adults’ subjective well-being (positive affect and depression) and to examine whether being a recipient of institutional care moderates these effects.

**Methods:**

Social support (provided and received), positive affect, and depressive symptoms were assessed twice (at baseline and 1 month later) for 277 older adults (age 77.39 ± 9.20 years, 67.50% women, 65% residents of an institutional care facility).

**Findings:**

Two structural equation models were analyzed: cross-sectional (at baseline) and longitudinal (after 1 month). The first model revealed a significant positive relationship between providing and receiving support and positive affect, and a negative relationship between receiving support and depression. However, being a recipient of institutional care appeared to be a significant moderator in the longitudinal model. Specifically, the findings indicated effects of both providing and receiving support on positive affect but only for noninstitutionalized older adults.

**Discussion:**

Although both types of support may be beneficial for older adults, their effects depend on the nature of social exchange and the dimensions of well-being. This suggests that such factors should be systematically investigated in future research.

## Introduction

Provided and received social support may have distinct consequences for individual well-being, as reflected in both theoretical developments and accompanying empirical findings. Equity theory assumes that receiving support may result in distress and guilt when it violates the reciprocity principle of social exchange; that is, when more support is received than given [1]. In contrast, according to social exchange theory, received support should be associated with greater well-being, as people strive to maximize their profits (receiving support from others) and minimize their losses (using up resources while supporting others) [2]. Finally, the esteem enhancement hypothesis suggests that supporting others is an antecedent of well-being increment [3,4].

Given these contrasting theories, it is no surprise that the results of research in this area have been incoherent. There has been more research on received support, and results show that it has a positive effect on well-being [5,6], no effect [7], or even side effects [8]. Although fewer studies have focused on provided support, the findings suggest that this type of support may have more beneficial effects than received support [9].

The effects of support in older adults are unclear too. Although some research indicates that received support is positively related to quality of life [10,11], depression [12,13], and distress [14], other research indicates that these relations can be negative [15,16]. This suggests that there are moderating factors that contextually modify the relationship between support and well-being. In addition, this relationship may vary depending on whether positive or negative dimensions of well-being are studied.

Those studies that have focused on both received and provided support suggest that they both have a beneficial effect on older adults’ well-being, but that the effect of provided support is stronger [17,18]. Interestingly, Warner et al. [19] did not confirm these findings. In their study, only providing support to others was positively related to quality of life; receiving support was negatively related to quality of life. In contrast, Li et al. [20] reported that overbenefited friendships are more strongly associated with life satisfaction than reciprocal friendships, which indicates a crucial role for received rather than provided support. However, as all these studies are cross-sectional, an alternative explanation that greater well-being promotes a coherent perception of social exchange cannot be excluded.

The results of longitudinal studies indicate that relations between social support and well-being are probably reciprocal. For example, in a community sample of people aged over 70 years, low support led to greater distress 22 months later and, within the same time lag, greater distress led to less received support [21]. Interestingly, distress appeared to be more stable than support, although for both, more variance was explained by change within time rather than by stability. This further suggests that the context of evaluation, especially the intervals between measurements, affects the results. Therefore, received support is likely to have temporary positive effects, while negative social interactions (i.e., criticizing, lack of sympathy and care) are related to long-term consequences for well-being [22]. In addition, the duration of provided support has distinct effects for the provider’s well-being: short-term support was negatively and long-term support positively related to depression [15]. Finally, one study indicates that the mortality risk among older adults was reduced by provided support and slightly increased by received support within 5 years, after controlling for possible confounders [23].

However, it is too early to draw many conclusions from these findings, as there are few studies of this kind. Additionally, most studies focus on negative, rather than positive, indices of adjustment, and thus tend to be pathogenically oriented. Finally, such studies only include samples of well-functioning older adults in their natural social surroundings. Demographic shifts, accompanied by changes in family structure [24], have resulted in increasing need for nursing and residential care [25], which makes research on social support in such institutions of special clinical importance.

### The present research

The present study aims to address these gaps. It includes both older adults living at home and residents of nursing homes. We hypothesize that this difference in conditions moderates the relationship between social support and well-being. In institutional care, the support exchange, on one hand, is regulated by well-defined social roles (i.e., resident and caregiver), but on the other hand it can be facilitated by reducing social isolation [26]. Thus, as the social surroundings probably promote being a recipient of support, this is a provided support that may contribute substantially to well-being in such a context. Two mechanisms can underlie this effect. First, being a provider of support may be an exceptional behavior, compared with being a recipient of support given by others, which is perceived not only as typical but also as obligatory for institutional care. Therefore, habituation is likely to occur. Second, as a violation of the reciprocity principle results in poorer well-being (according to equity theory), provided support may act as a protective factor, even if an individual’s supportive behaviors are not present objectively, but only overestimated, to balance the ongoing social interactions.

In this study, well-being is defined in terms of positive affect (PA) and depressive symptoms; current findings indicate that over longer time periods these should be treated as two independent unipolar dimensions of affective functioning [27], including among older adults [28]. Therefore, social support may have distinct effects on these dimensions; tested in a systematic way, this could clarify the mixed research findings reported so far.

Finally, this study’s longitudinal design captures cross-sectional relations as well as the delayed relations that can serve as a proxy to explore the stability of the effects of support. There are reasons to expect that the effect of provided support may last longer than the effect of received support. Provided support requires more autonomous activity than being just a recipient of others’ supportive behaviors and it is connected with positive changes in self-evaluations [29,30]. Thus, bolstered personal resources can maintain well-being beyond the period of support provision.

To sum up, the aim of the current study is twofold:

1. To examine the cross-sectional and longitudinal effects of provided and received support on well-being, described by two affective dimensions.
2. To examine whether being a recipient of institutional care is a moderator of the abovementioned effects.

## Materials and Methods

### Participants and procedure

Participants were 277 elderly people (67.5% women) aged 60–100 years (*M* = 77.39, *SD* = 9.20), assessed twice: at baseline (T1) and 1 month later (T2; *N* = 212). Of the participants, 65% (T_1_ = 180; T_2_ = 138) were residents of nursing homes (range of stay from 1 to 303 months, *M* = 61.93 months, *SD* = 59.84) and 35% (T_1_ = 97; T_2_ = 74) attended daily seniors clubs (for about 5–6 hours each day). Inclusion criteria were as follows: age ≥60, lack of cognitive disorders (no diagnosis of dementia or mild cognitive impairments, and efficient cognitive functioning confirmed by the care facility personnel), and lack of serious acute somatic illness that may significantly affect the results. Most participants were single (unmarried, widowed, divorced; 83%), with secondary or primary education (71%), had not used medical care for the previous 6 months (63%), and described their material status as average (62%). Participants suffered from more than four coexisting chronic illnesses (0–12; *M* = 4.53, *SD* = 2.63) and took on average about six medicines per day (*M* = 6.42, *SD* = 4.47). The average functional status of participants was relatively high: the mean score on the Katz Index of Activities of Daily Living (ADL) [31] was 5.65 (*SD* = .88, range = 0–6, Cronbach’s α = .71), and on the Lawton Instrumental Activities of Daily Living (IADL) Scale [32] was 20.89 (*SD* = 3.88, range = 8–24, Cronbach’s α = .85). Subjectively assessed health status, measured by two items (“Overall, my current health is…” and “Compared with people of my age and sex, my current health is…”) rated on a five-point Likert scale from 1 (*poor*) to 5 (*excellent*), was perceived as acceptable (*M* = 2.54, *SD* = .87, Cronbach’s α = .61).

The institution did not differentiate the participants in terms of controlled variables, except for age, functional status, subjective health, and the number of administered drugs. The residents of nursing homes (*M*_NH_ = 79.14, *SD* = 9.07) were significantly older than those attending the daily seniors clubs (*M*_SC_ = 74.15, *SD* = 8.60, *t*_275_ = −4.44, *p* < .001), rated both their health (*M*_NH_ = 2.44 ± .85 vs. *M*_SC_ = 2.72 ± .87, *t*_275_ = 2.62; *p* = .009) and functional status (ADL: *M*_NH_ = 5.52 ± 1.04 vs. *M*_SC_ = 5.90 ± .30, *t*_229.67_ = 4.54, *p* = .001; IADL: *M*_NH_ = 19.84 ± 4.12 vs. *M*_SC_ = 22.82 ± 2.41, *t*_273.15_ = 7.57; *p* < .001) as worse, and took significantly more drugs daily (*M*_NH_ = 6.78 ± 4.33 vs. *M*_SC_ = 5.57 ± 4.15, *t*_271_ = 2.22; *p* = .050).

The study protocol was evaluated and approved by the University of Social Sciences and Humanities ethical committee in Warsaw. Written informed consent was obtained from all participants. Participation in the study was voluntary. At both measurement points, participants completed questionnaires to evaluate social support, affect, depressive symptoms, and health indicators. Taking into consideration the specificity of the study group, abbreviated or experimental versions of the research tools were used (i.e., consisting of 2–6 items).

The sample attrition analyses indicated that data missingness was similar in the nursing home and senior club groups (approximately 23%); respondents and non-respondents did not differ on sociodemographic and medical variables except age (non-respondents were older than respondents; χ^2^_1_ = 16.23, *p* < .001, odds ratio = .94). Of the main study variables, significant differences were found only for provided support, which was higher for non-respondents than for respondents, χ^2^_1_ = 6.53, *p* = .011, odds ratio = .62, *M* = 2.89 ± .75 vs. *M* = 2.61 ± .78.

### Measures

#### Social support

Received support was measured using six items selected from the Berlin Social Support Scales (BSSS) [33]. This scale’s internal consistency ranged from α = .92 at T1 to α = .93 at T2. Provided support was measured using three questions phrased congruently with the BSSS received support items (“I comforted her/him or gave her/him encouragement,” “I gave her/his advice or information,” “I offered her/him help”). This scale’s internal consistency ranged from α = .82 at T1 to α = .84 at T2. All statements were assessed on a four-point scale from 1 (*strongly disagree*) to 4 (*strongly agree*). The indicators of received and provided support were the arithmetic means of the answers. Higher scores indicate greater levels of support. For the purposes of this study, only values from the first measurement were used.

#### Positive affect

Positive affect (PA) was measured twice using three items (joy, satisfaction, optimism) from the Positive and Negative Affect Schedule (PANAS) [34]. Participants rated how they currently felt on a seven-point scale ranging from 1 (*not at all*) to 7 (*very strongly*). Internal consistency ranged from α = .79 at T2 to α = .82 at T1. Higher scores indicated greater PA.

#### Depressive symptoms

Symptoms of depression were evaluated twice with 11 items from the Centre for Epidemiological Studies Depression Scale (CES-D) [35], which were assessed using a four-point scale ranging from 0 (*rarely or never*) to 3 (*often*). Higher scores indicate greater depressive symptoms. Internal consistency was α = .77 at T1 and T2.

### Data analyses

**Fig 1:**
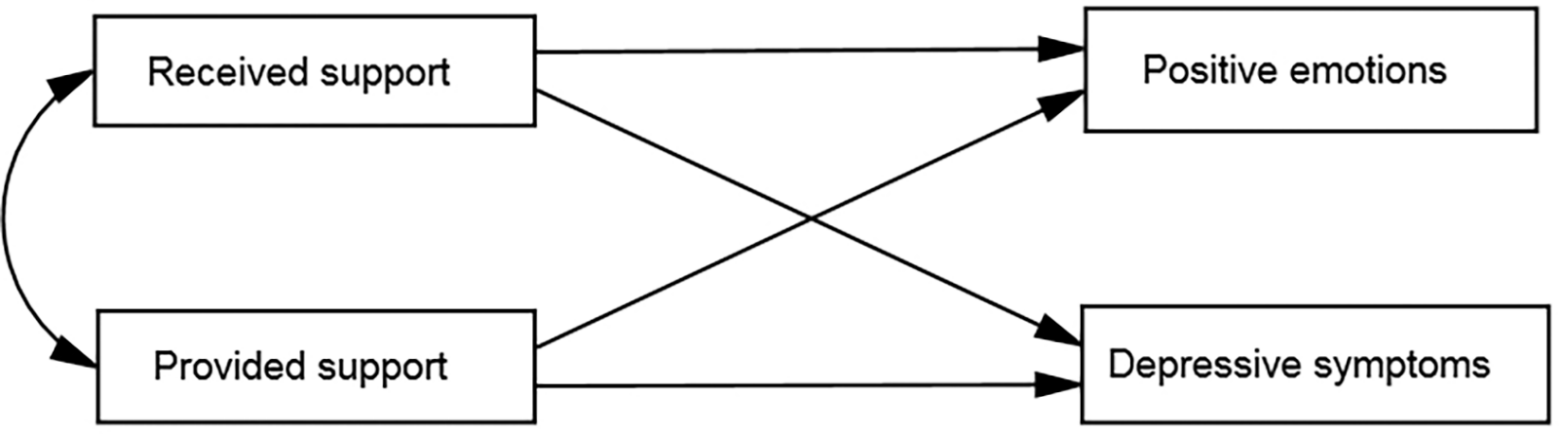
Model of relationships between received and provided social support and well-being.

Analyses were conducted using IBM SPSS (IBM Corp.; Armonk, NY) and AMOS, both versions 22. Structural equation modeling was used to examine the relations between received and provided support and well-being (i.e., PA and depressive symptoms) (see Fig 1). Two models were tested: a cross-sectional model for the relationship between support and well-being at baseline (T1), and a longitudinal model for the time effect (T1 → T2) of support, after controlling for the baseline level of well-being. For this purpose, regression standardized residuals were obtained, where values of the well-being variables at T2 were regressed on the baseline values of the same variables [36]. These residuals were then included in the structural equation modeling analyses as well-being variables in the longitudinal model. The data showed a multivariate normal distribution. The sociodemographic variables: age, gender, marital status, subjective and objective (number of diseases) health, as well as functional status (ADL, IADL), were entered into the joint models for both samples as covariates, however in the final models only those with paths significantly different from zero were included. To maximize the utilization of the data, we used multiple imputation of residuals [37] using all data in the tested models. The comparison between original (*N* = 212) and imputed (*N* = 277) data sets revealed no significant sample differences on analyzed variables. Thus, the results from the imputed file were reported.

Following the recommendations of Hooper, Coughlan, and Mullen [38], goodness of fit for the models was based on (1) the chi-square value (χ^2^), (2) the normed chi-square (χ^2^*/df*), (3) the non-normed fit index (NNFI, also known as Tucker–Lewis Index, TLI), (4) the comparative fit index (CFI), (5) the adjusted goodness-of-fit index (AGFI), and (6) the root mean square error of approximation (RMSEA). An insignificant χ^2^ test indicates a good model fit, although its use is not free from limitations, especially for small sample sizes. Thus, the ratio of χ^2^ to degrees of freedom was also used, with values lower than 2 assumed to be satisfactory [36]. The NNFI, CFI and AGFI threshold values of .90 indicate satisfactory fit, whereas values above .95 indicate good fit. For a well-fitting model, the RMSEA should be close to 0: values below .05 indicate a good model fit and a value of .08 represents reasonable errors of approximation. After establishing a model with a satisfactory goodness of fit, multiple group analysis was conducted to test for possible moderation effects from institutional care (nursing homes vs. seniors clubs). The goodness of fit of both constrained (i.e., assuming all regression coefficients are equal across groups) and unconstrained (assuming all the parameters were freed across groups) models were tested. Delta chi-square test (Δχ^2^), the Akaike information criterion (AIC), and ΔAIC were computed to compare models.

## Results

### Descriptive statistics

Descriptive statistics and correlations for nursing home and seniors club samples are presented in Table 1. There were moderate correlations between depressive symptoms and PA and low correlations between well-being and social support. PA decreased over time (Wilks’ lambda *F*_1,137_ = 23.19, *p* < .001, partial eta^2^ = .14), whereas depressive symptoms increased over time (Wilks’ lambda *F*_1,137_ = 4.94, *p* = .028, partial eta^2^ = .04), but only for nursing home residents. For the seniors club participants, PA and depression were stable during the study period, Wilks’ lambda *F*_1,73_ = .51, *p* = .478; Wilks’ lambda *F*_1,73_ = .67, *p* = .417, respectively. Additionally, the nursing home group showed less received support (*F*_1,229.13_ = 3.93, *p* = .049), lower PA (T1: *F*_1,276_ = 4.10, *p* = .044; T2: *F*_1,211_ = 17.07, *p* < .001), and greater depressive symptoms (T1: *F*_1,238.24_ = 10.85, *p* = .001; T2: *F*_1,211_ = 10.54, *p* = .001) compared with the seniors club participants.

**Table 1:** Descriptive statistics and bivariate correlations.

	*M*_*NH*_ *(SD)*	*M*_*SC*_ *(SD)*	1.	2.	3.	4.	5.	6.
**1. Rec**_**1**_	3.09 (.95)	3.30 (.78)	-	.35**	.32**	.23**	−.19**	−.12
**2. Prov**_**1**_	2.68 (.80)	2.67 (.73)	.23*	-	.29**	.28**	−.19**	−.15
**3. PA**_**1**_	12.32 (4.96)	13.53 (4.38)	.20*	.27**	-	.64**	−.57**	−.51**
**4. PA**_**2**_	10.48 (4.69)	13.17 (4.18)	.29*	.39**	.77**	-	−.51**	−.60**
**5. CES-D**_**1**_	22.57 (6.47)	20.34 (5.11)	−.31**	−.14	−.64**	−.60**	-	.73**
**6. CES-D**_**2**_	23.35 (6.05)	20.58 (5.78)	−.15	−.23*	−.47**	−.60**	.68**	-

Rec, receiving social support; Prov, providing social support; PA, positive affect; CES-D, depressive symptoms; NH, nursing homes; SC, seniors clubs; Index 1, 2, time 1, 2, respectively.

T1: N_NH_ = 180; N_SC_ *=* 97; T2: N_NH_ = 138; N_SC_ *=* 94.

Coefficients for the nursing homes are shown in the upper half of the table, and those for seniors clubs in the lower half.

*p < .05

**p < .01.

### Social support and well-being: Cross-sectional and longitudinal analysis

**Fig 2:**
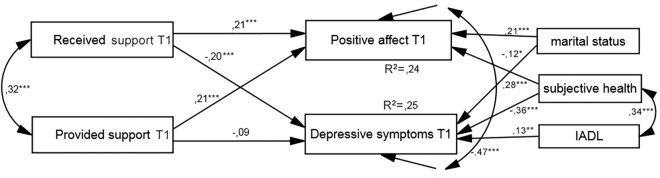
Resultant model of cross-sectional relationship between social support and well-being for both samples. IADL, Lawton Instrumental Activities of Daily Living (higher scores indicate greater independence); values presented are standardized coefficients, ^*^p < .05, ^**^p < .01, ^***^p < .001.

The cross-sectional model of the relationship between social support and well-being fitted the data well (see Table 2). The effects of received social support on both PA and depressive symptoms were statistically significant and in different directions (see Fig 2). However, provided support had a significant effect only on PA. In addition, there were significant paths leading from marital status and subjective health to both dimensions of well-being, whereas functional status was positively and weakly related only to depression.

**Table 2:** Cross-sectional and longitudinal model fit indicators: structural equation modeling results.

Model	χ^2^	*df*	*p*	χ^2^/*df*	NNFI	CFI	AGFI	RMSEA (90% Cl)
**Cross**	16.26	9	.062	1.81	.943	.976	.951	.054 (.00; .09)
**Time**	5.39	5	.370	1.08	.99	.99	.973	.02 (.00; .08)

Cross, cross-sectional model; Time, longitudinal model; χ^2^/df, normed chi-square; NNFI, non-normed fit index (Tucker–Lewis index); CFI, comparative fit index; AGFI, adjusted goodness-of-fit index; RMSEA, root mean square error of approximation; 90% Cl, 90% confidence intervals.

**Fig 3:**
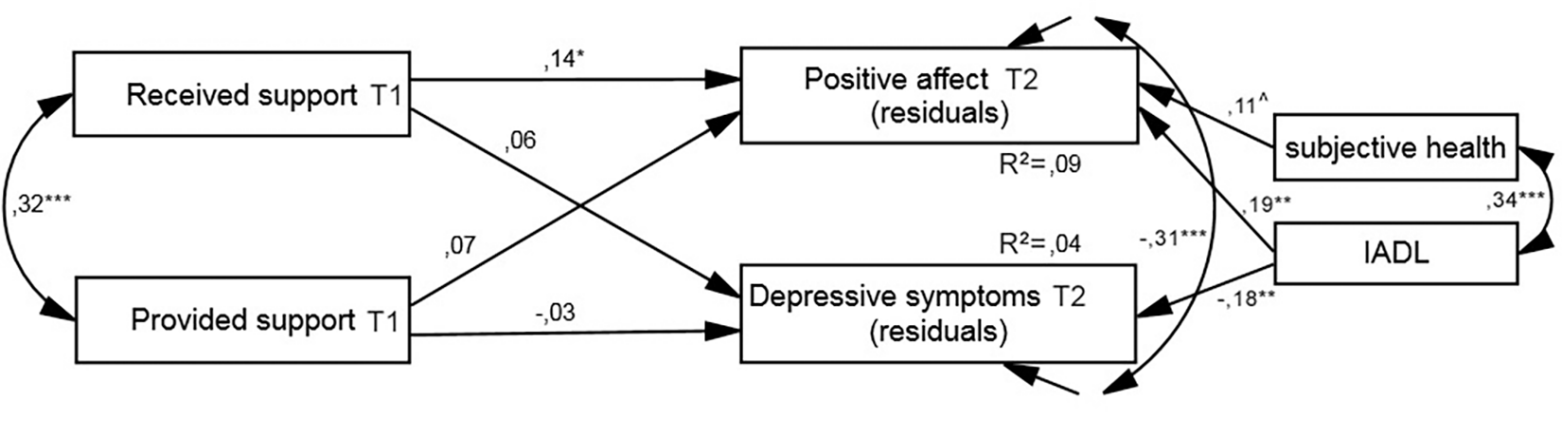
Resultant model of time effect of social support on well-being for both samples. IADL, Lawton Instrumental Activities of Daily Living (higher scores indicate greater independence); values presented are standardized coefficients, ^*p* < .07, ^*^p < .05, ^**^p < .01, ^***^p < .001.

The time effect model also fitted the data well (see Table 2). However, the only significant effect was that of received support on PA: the greater the support received from others, the greater the PA 1 month later, after controlling for its baseline level (see Fig 3). In addition, the effect of functional status on PA and depression was significant.

### Moderation effects of institutionalization

Cross-sectional models with institutionalization as a grouping variable demonstrated a good fit (see Table 3). A model comparison (unconstrained vs. constrained) did not indicate significant differences between models (Δχ^2^ was insignificant). The model with equal regression coefficients across groups (constrained) was more parsimonious and thus appeared to fit the data better. Therefore, a moderation effect of institutionalization was not supported.

**Table 3:** Moderation effect of institutionalization: group comparison.

Model	χ^2^	*df*	*p*	χ^2^/*df*	NNFI	CFI	RMSEA (90% Cl)	AIC
**Cross-sectional model**								
**Unconstr.**	23.99	18	.155	1.33	.95	.98	.03 (.00; .07)	127.99
**Constr.**	36.20	27	.111	1.34	.95	.97	.03 (.00; .06)	122.20
Δχ^2^ = 12.21, Δ*df* = 9, *p* = .202, ΔAIC = 5.79								
**Longitudinal model**								
**Unconstr.**	17.31	10	.068	1.73	.82	.94	.05 (.00; .09)	105.31
**Constr.**	36.92	17	.003	2.17	.72	.84	.06 (.04; .09)	110.92
Δχ^2^ = 19.61, Δ*df* = 7, *p* = .006, ΔAIC = 5.61								

Unconstr., unconstrained model; Constr., constrained model; χ^2^/df, normed chi-square; NNFI, non-normed fit index (Tucker–Lewis Index); CFI, comparative fit index; RMSEA, root mean square error of approximation; 90% Cl, 90% confidence intervals; AIC, Akaike information criterion.

**Fig 4:**
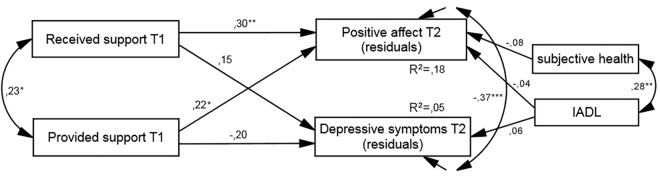
Resultant model of time effect of social support on well-being for seniors club participants. IADL, Lawton Instrumental Activities of Daily Living (higher scores indicate greater independence); values presented are standardized coefficients, ^*^p < .05, ^**^p < .01, ^***^p < .001.

**Fig 5:**
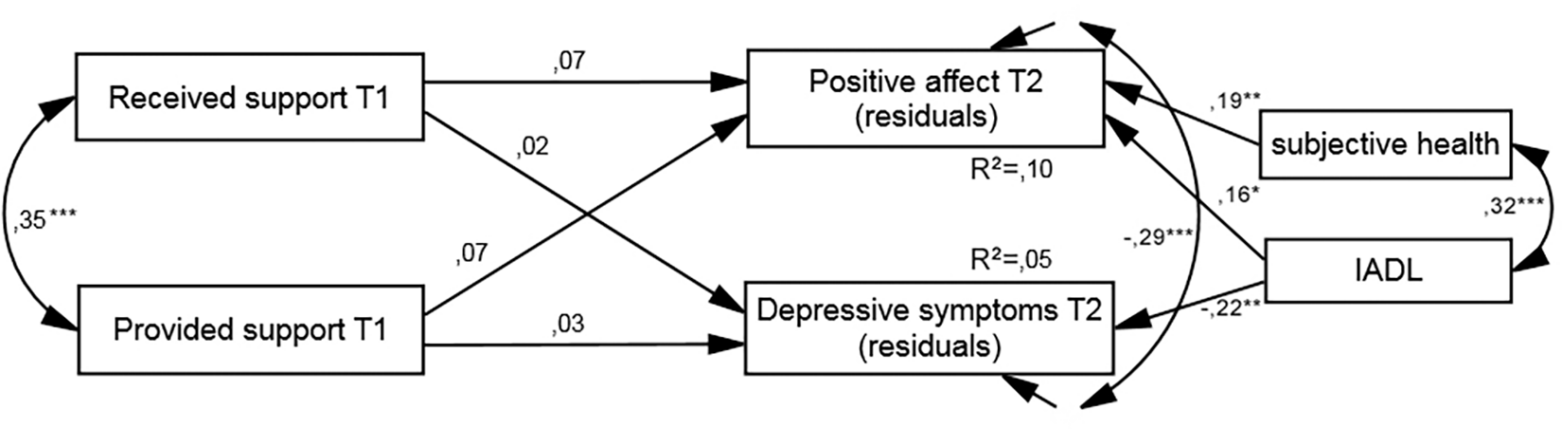
Resultant model of time effect of social support on well-being for nursing home participants. IADL, Lawton Instrumental Activities of Daily Living (higher scores indicate greater independence); values presented are standardized coefficients, ^*^p < .05, ^**^p < .01, ^***^p < .001.

In the longitudinal model, multiple group analysis indicated significant differences between unconstrained and constrained models (significant Δχ^2^, see Table 3). Both models (unconstrained and constrained) had acceptable fit indicators, but the unconstrained model showed better values. The comparison of models, expressed by significant Δχ^2^, indicated a difference between their goodness of fit, which led us to reject the constrained model. Thus, institutional care appeared to be a moderator of the longitudinal relationship between social support and well-being. Specifically, the model showed that for the seniors club participants both the receiving and providing of social support was a positive predictor of PA 1 month later (see Fig 4). The effect of social support on depression was not significant. Covariates were unrelated to well-being in this group. In contrast, for the nursing home group, only the covariates were related to well-being; there were no longitudinal effects of social support (see Fig 5).

## Discussion

The aim of the study was to examine relations between support and well-being from two temporal perspectives: cross-sectional and longitudinal, with a 1-month lag between measurement points. Both models revealed only one significant correlation: a relation between received support and PA. The greater the received support, the greater the PA. However, the moderation analysis indicated that the effect obtained in the longitudinal model can be entirely attributed to the participants from the seniors clubs, as among nursing home residents all the relations between support and well-being turned out insignificant.

These patterns differed from those revealed by the cross-sectional model, which showed the same relations for both groups. Namely, the received support was associated with both greater PA and less depression, whereas given support was not significantly related to depression, but was positively related to PA. Although the cross-sectional nature of the model prevents any inferences about the causality of these relations, they are in accord with the generally beneficial consequences of received support that are repeatedly observed in research as direct effect of support [39]. They also suggest that being a support provider is related to PA but not necessarily related to depressive symptoms, which has also been reported in a previous study [40].

Interestingly, the cross-sectional results were the same for both groups, even though the groups differed significantly on given support and well-being: the nursing home participants reported lower levels of support and well-being. Thus, the transient effects of support seem relatively context-free, but the postponed effects are probably more prone to such influences. There are at least three non-exclusive explanations of the longitudinal between-group differences. First, as was mentioned in the introduction, patients of nursing homes may get used to higher levels of support and perceive this as normal because of the nursing character of these homes [41]. Therefore, from a longer perspective, received support does not contribute greatly to their well-being. Additionally, they may perhaps perceive as supporting only those interactions exceeding the scheme of support exchange in a given institution or involving special, more spontaneous, and voluntary sources of this support (e.g., other pensioners, visiting relatives) [42]. However, such support, as it stems from interactions other than typical social exchanges, may be relatively rare and have a time-limited effect. Second, depression symptoms among nursing home residents may be too high for such institutional support to reduce them effectively. Finally, it may not be the intensity of symptoms that is crucial here, but their origin [43]. Even though the proxy of objective somatic health (expressed by number of diseases and functional state) was controlled in the models, a somatogenic etiology of depression symptoms [44] may have affected the results, limiting the corrective potential of any kind of support.

The different mechanisms underlying well-being in both groups are also indicated by the dynamics of well-being during the study period. PA decreased in nursing home residents, whereas symptoms of depression increased between the measurement points. Hence, both dimensions are negatively, though moderately, correlated (see Table 1). For the seniors club participants, levels of both well-being dimensions were stable. Thus, the relation between PA and depressive symptoms in non-clinical groups of older adults is dynamic and may be dependent on the social setting in which they live. In addition, although the lower affective well-being of nursing home residents reflects the findings of other studies [45,46], our study reveals that baseline support, regardless of its character, does not prevent their well-being *reducing* over time.

Finally, contrary to the assumptions of equity theory, both types of support were only weakly correlated (i.e., greater received support was not systematically accompanied by greater provided support). However, as in all studies using self-descriptive measures of social support, the lack of verification of participants’ declarations with actual behavior is a substantial limitation. Moreover, we did not analyze the relationship between the *change* of support intensity and the *change* of well-being. Neither did we take into account possible differences arising from the type of functional support. The very few studies on given emotional, instrumental, or informative support show that their effects on the support provider’s well-being may be dependent on what kind of support is given [47], who it is given to [48], and for how long [49]. The present results are in accord with previous work on received support, which indicates that different support sources modify the effectiveness of received support type [50]; this could help to explain the results obtained for nursing home participants. Thus, this issue requires further exploration. It should be also emphasized that there were different numbers of participants in each group and that the groups may be highly specific; the statistical control of sociodemographic and health-related variables does not take into consideration the mechanisms underlying such differences. For instance, people in nursing homes differ from people from seniors clubs in many sociodemographic and functional aspects; perhaps their ability to build social network is also different: participants of seniors clubs may be more active and autonomous in satisfying their social needs.

Summing up, the findings indicate that the incoherence of studies on the relationship between support and well-being may be caused by different time lags between measurement of support and its potential effects, the valence of these effects (i.e., whether they are positive or negative), and the broader social context of participant functioning, which is not always directly included in the study design. Moreover, both received and provided support seem to be important to the positive aspect of well-being, but longitudinally only for well-functioning older people. Although the percentage of explained variance for each model is low, it is similar to values reported in other studies on this subject [50,51]. Additionally, because of the control of baseline values, this may be interpreted as incremental variance due to social support (and covariates), which may be an additional contribution of the study. However, the meaning of these findings is not entirely clear. The results could be interpreted as supporting the esteem enhancement theory; however, the benefits from received support appear to confirm social exchange theory.

Finally, neither given nor received support prevented the reduction of well-being in nursing home participants. On the contrary, these participants had lower ratings of received support than did noninstitutionalized participants. This may point to a mechanism similar to that described as support deterioration [52]. Interestingly, as our findings suggest, this may be associated not only with older age but also with the specifics of functioning in nursing institutions. This phenomenon requires further study.

## Supporting Information

S1 Data Dataset for “Well-being and Institutional Care in Older Adults: Cross-Sectional and Time Effects of Provided and Received Support” in.sav format. (SAV)
